# Integrated analysis of immune-related genes in endometrial carcinoma

**DOI:** 10.1186/s12935-020-01572-6

**Published:** 2020-10-02

**Authors:** Yiru Wang, Yunduo Liu, Yue Guan, Hao Li, Yuan Liu, Mengjun Zhang, Ping Cui, Dan Kong, Xiuwei Chen, Hang Yin

**Affiliations:** 1 The Department of Gynecologic Oncology, Harbin Medical University Cancer Hospital, Harbin, 150040 Heilongjiang China; 2 The Department of Radiotherapy Oncology, Harbin Medical University Cancer Hospital, Harbin, Heilongjiang China

**Keywords:** Long non-coding RNA, Immune, Biomarkers, Transcription factor, Endometrial cancer, The cancer genome atlas

## Abstract

**Background:**

Exploring novel and sensitive targets is urgent due to the high morbidity of endometrial cancer (EC). The purpose of our study was to explore the transcription factors and immune-related genes in EC and further identify immune-based lncRNA signature as biomarker for predicting survival prognosis.

**Methods:**

Transcription factors, aberrantly expressed immune-related genes and immune-related lncRNAs were explored through bioinformatics analysis. Cox regression and the least absolute shrinkage and selection operator (LASSO) analysis were conducted to identify the immune and overall survival (OS) related lncRNAs. The accuracy of model was evaluated by Kaplan–Meier method and receiver operating characteristic (ROC) analysis, and the independent prognostic indicator was identified with Cox analysis. Quantitative real-time polymerase chain reaction (qRT-PCR) were conducted to detect the accuracy of our results.

**Results:**

A network of 29 transcription factors and 17 immune-related genes was constructed. Furthermore, four immune-prognosis-related lncRNAs were screened out. Kaplan–Meier survival analysis and time-dependent ROC analysis revealed a satisfactory predictive potential of the 4-lncRNA model. Consistency was achieved among the results from the training set, testing set and entire cohort. The distributed patterns between the high- and low-risk groups could be distinguished in principal component analysis. Comparisons of the risk score and clinical factors confirmed the four-lncRNA-based signature as an independent prognostic indicator. Last, the reliability of the results was verified by qRT-PCR in 29 cases of endometrial carcinoma and in cells.

**Conclusions:**

Overall, our study constructed a network of transcription factors and immune-related genes and explored a four immune-related lncRNA signature that could serve as a novel potential biomarker of EC.

## Background

Endometrial cancer (EC) is considered as the most common gynecological malignancy [1]. The incidence and mortality of EC have shown an upward tendency annually worldwide, affecting approximately 63,000 new patients and contributing to over 11,000 deaths in the U.S. each year [2]. The increasing morbidity of EC in the United States and worldwide is particularly due to obesity, senility, early menarche, late menopause and Lynch syndrome [3]. Endometrial cancer cells are of glandular epithelial origin and are invasive [1]. Patients with low-degree, non-invasive and early-stage tumors frequently have a comparatively favorable prognosis [4]. In contrast, patients with advanced EC with high-grade histologic subtypes have worse survival [5, 6].

Therefore, strengthening the mechanism research of EC is of great clinical significance.

Long noncoding RNAs (lncRNAs) are longer than 200 nt in length and have no protein coding functions [7]. LncRNAs are a class of transcripts involved in the regulation of signal pathway activities by influencing protein-encoding gene expression [8]. Overwhelming evidence suggests that lncRNAs regulate gene expression by means of chromatin modification, transcriptional activation and transcriptional interference in the occurrence and development of tumors [9]. In addition, based on multiple relevant studies, some lncRNAs have been proven as biomarkers for early diagnosis and prognosis evaluation for their apparent cell and tissue-type specificity [10]. As our previous study found, novel 11-lncRNA have been identified as the prognostic factor of head and neck squamous cell carcinoma [11], and a 9-lncRNA signature has been suggested as an independent prognostic indicator to predict survival in clear cell renal cell carcinoma [12]. Nevertheless, studies of lncRNAs in EC remain rare. Therefore, exploring key EC-related lncRNAs to predict survival and systematically clarifying their functions and clinical significance in EC are indispensable.

The immune system plays a significant role in the process of tumorigenesis, tumor development and metastasis [13]. In the last 3 years, the clinical application of immunotherapies in oncology has gained considerable attention showing promising progress. To date, immunotherapy with checkpoint inhibitors has been demonstrated to have the ability to improve gynecologic cancer clinical trial outcomes [14]. For instance, the positive modulation of adaptive immunity through anti-PD-1 or anti-PD-L1 antibodies promotes T cell proliferation, enhances the anti-tumor effect of T cells and represses the immune escape of cancer cells, ultimately resulting in gynecological tumor regression [15]. Most previous researches have concentrated on the function of proteins in this progression, but further research on the specific functions of RNAs is still relatively scant [16]. LncRNAs are emerging as regulatory complexes that influence gene expression and pathways in the modulation of the immune system [17]. In addition, transcription factors (TFs) have been reported to trigger dynamic changes in immune cells to a certain degree and participate in the regulation of the immune response [18, 19].

Nevertheless, the clinical implication of immune-related lncRNAs in endometrial cancer prognostication has not been well investigated. Hence, it is crucial to confirm the complete landscape of lncRNAs that are engaged in the regulation of the immune response. LncRNAs in the context of the immune system may be of great significance not only in providing reasonable methods for immunotherapy but also in offering accurate therapeutic options for tumors.

In the current study, we explored transcription factors and immune-related genes from the TCGA database. Then, immune-related lncRNAs were explored. Further, we found the four-lncRNA could predict survival and serve as biomarker. Kaplan–Meier survival analysis and time-dependent ROC analysis revealed the satisfactory predictive potential of the model. Consistency was achieved among the results from the training set, testing set and entire cohort. The distributed patterns between the high- and low-risk groups can be distinguished in principal component analysis. Next, comparisons of the risk score and clinical factors confirmed the four-lncRNA-based signature as an independent prognostic indicator. We compared the accuracy of our signature with other existing predictive models, and explored the four lncRNAs separately in database. Finally, we validated gene expression levels in clinical samples and cell lines by qRT-PCR.

## Methods

### Data collection and differential expression analysis

The clinical data including age, stage and survival status were obtained from The Cancer Genome Atlas (TCGA) database (https://portal.gdc.cancer.gov/). Patients whose clinical characteristic information was incomplete were excluded. In total, 541 endometrial cancer patients were enrolled in our study. The gene data were derived from the TCGA. The gene sequence data of 552 EC tumor tissues and 23 normal tissues were collected. And Differential expression (DE) analysis of genes was carried out by R software limma package. In our research, the criteria for identifying differentially expressed genes were |Log2FC|> 1 and FDR < 0.05 (FC, fold change; FDR, false discovery rate).

### Differentially expressed transcription factors and immune genes

Data on 318 transcription factors (TFs) was obtained from Cistrome (https://cistrome.org/) [20], and the list of 2498 immune genes was downloaded from ImmPort (https://immport.niaid.nih.gov) [21]. Next, differentially expressed TFs were extracted from the overlap between TFs and all of differentially expressed genes (DEGs). And we acquired the immune-related DEGs in the same way. Then, the immune-prognosis-associated DEGs were screened by using univariate Cox regression model of immune-related DEGs. The Cox regression analysis subjected was conducted in survival package of R. Cytoscape 3.7.2 was applied to visualize the interaction network of TFs and immune-OS-related DEGs [22].

### Identification of immune-related lncRNAs

The co-expression analysis can deeply reflect the expression regulation relationship between genes. We obtained the immune-related lncRNA associated with prognosis by co-expression analysis of immune-OS-related DEGs and lncRNAs. And the correlation analysis was conducted by the limma package for R. According to the analysis results, immune-related lncRNAs (|cor|> 0.4, P < 0.001) were screened for follow-up study.

### Functional enrichment analysis

We explored the Gene Ontology (GO) and KEGG pathways with the clusterProfiler package to elucidate the molecular functions and cellular components of the differentially expressed genes.

### Identification of prognostic lncRNAs

All 541 patients were randomly divided into a training set (272 patients) and a testing set (269 patients). Then, univariate and multivariate Cox regression analyses were performed. The risk score was an indicator of predicting an EC patient’s prognostic risk and that of each individual sample was computed with the formula shown in Additional file 1: Figure S1. All individuals were divided into high- and low-risk groups according to differentially expressed genes in the high- or low-risk score. The overall survival (OS) in the high- and low-risk groups was compared by Kaplan–Meier survival curves. Receiver operating characteristic (ROC) analysis was used to assess the accuracy and diagnostic value of the lncRNA risk model.

### Cell culture

The normal human endometrial epithelial cell line hEEC and human endometrial adenocarcinoma cell lines HEC-1A and Ishikawa were obtained from Heilongjiang Cancer Institute (Harbin, China). All cell lines were cultured in Dulbeco’s modified eagle medium (DMEM) supplemented with 10% fetal bovine serum (FBS) and maintained at 37 °C with 5% CO2.

### Collecting EC samples and performing quantitative real-time polymerase chain reaction (qRT-PCR) verification

After obtaining the approval of the ethics committee of Harbin Medical University, we prospectively collected 29 pairs of endometrial cancer tissues and normal tissues from patients in the Gynecology Department of the Cancer Hospital of Harbin Medical University, between January 2019 to December 2019. All the participants signed informed consent forms (Additional file 2: Figure S2). All patients were diagnosed with primary endometrial cancer and did not receive adjuvant or neoadjuvant therapy before surgery, but patients with high-risk factors received postoperative radiotherapy. The characteristics of patients were listed in Table 1. All tissue specimens were quickly frozen in liquid nitrogen and stored at − 80 °C. Total RNA was extracted from the sample using TRIzol reagent (Invitrogen, Carlsbad, CA), and then reverse transcription and PCR reactions were performed using ReverTra Ace qPCR RT-PCR kit (Toyobo, Shanghai). All RNA‐primers were obtained from Generay Biotech (Shanghai, China). The sequences of applied primers were listed in Additional file 3: Table S1. Results were normalized to β-actin and calculated through 2^−ΔΔCt^ method. The detailed information of this experiment was the same as our previous study [23, 24], and all steps were carried out according to the manufacturer’s instructions.

**Table 1:** The clinic-pathological factors of EC patients

Characteristics	n	FP671120.4		P	LINC02381		P	LNCTAM34A		P	AC074212.1		P
	29	Low (n = 14)	High (n = 15)	value	Low (n = 14)	High (n = 15)	value	Low (n = 14)	High (n = 15)	value	Low (n = 14)	High (n = 15)	value
Age(year)													
< 60	20	11	9	0.280	7	13	0.033	9	11	0.599	10	10	0.782
> 60	9	3	6		7	2		5	4		4	5	
Histological grade													
G1	14	10	4	0.047	11	3	0.006	7	7	0.535	4	10	0.096
G2	14	4	10		3	11		6	8		9	5	
G3	1	0	1		0	1		1	0		1	0	
Figo stage													
I	24	11	13	0.564	13	11	0.164	12	12	0.684	13	11	0.286
II–III	5	3	2		1	4		2	3		4	1	

### Statistical analysis

The differentially expressed genes between tumor and normal tissues were analysed by R software package “limma”. Cox correlation analysis was used to confirm the relationships between the selected 4-lncRNA and patient outcomes. Survival curves were drawn with the survival package for R. The distributed patterns of high- and low-risk subsamples were described by carrying out principal component analysis (PCA). P < 0.05 was regarded to indicate statistically significant differences.

## Results

### The exploration of immune-related and differently expressed genes and TFs in EC

**Fig. 1:**
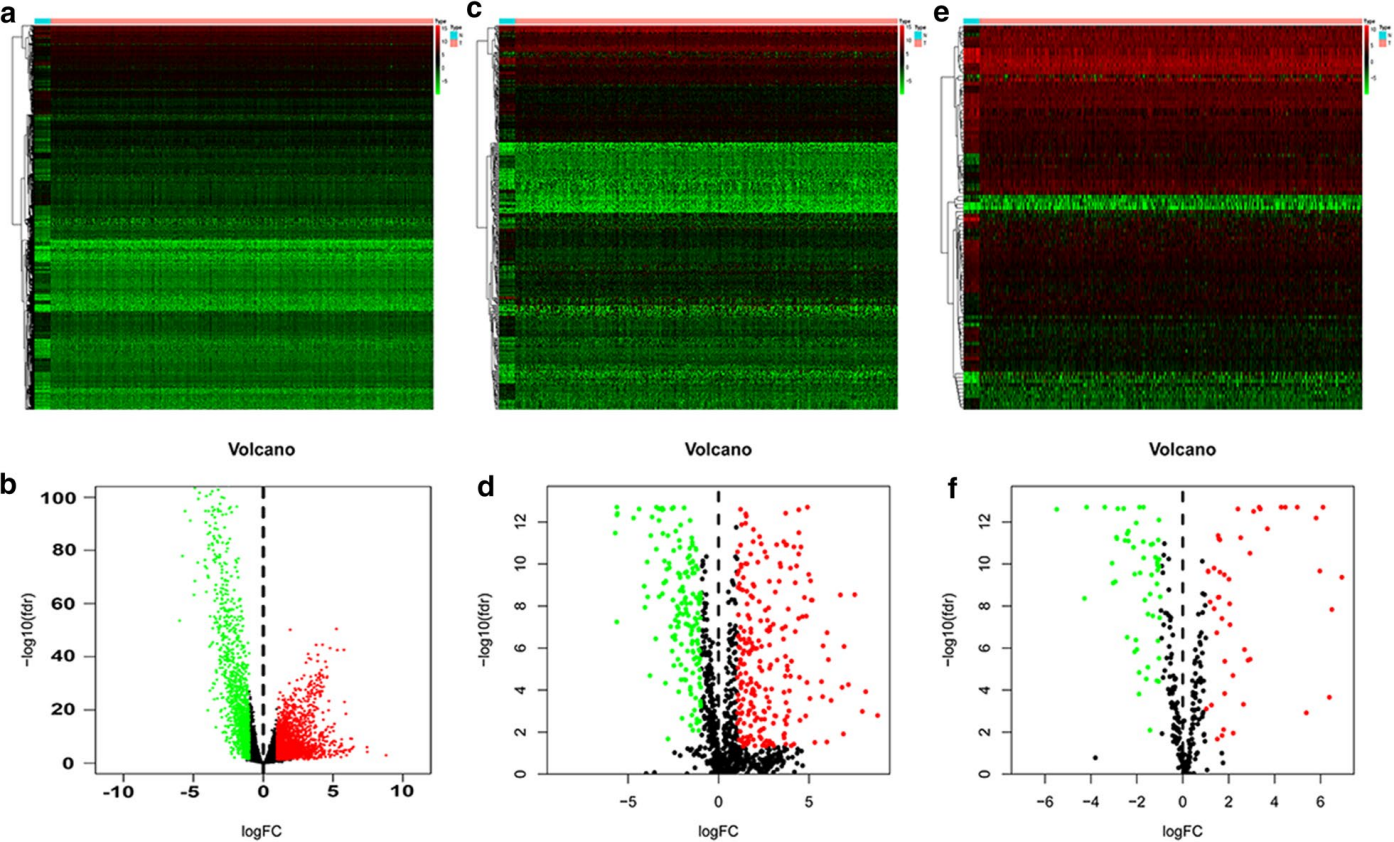
Heatmap and volcano plot show the different expressions of genes between 552 tumor tissues and 23 normal controls in endometrial cancer (EC). The green or red plot represented down-or up-regulation of genes respectively. **a**, **b** DE genes. **c**, **d** DE immune-related genes. **e**, **f** DE transcription factors. *DE* differentially expressed

Here, we identified 6267 DE genes (3861 upregulated genes and 2406 downregulated genes) between tumor and normal tissues (Fig. 1a, b). Afterwards, we collected 2498 immune-related genes. The results of differential gene expression analysis showed that 410 immune-relevant genes were differentially expressed in EC (Fig. 1c, d). The prognostic association between the 410 DE genes and patient OS was estimated by univariate Cox analysis, and 53 immuno-OS-related DE genes with a P-value < 0.05 were distinguished for the following analysis (Additional file 4: Figure S3). A total of 102 TFs (55 upregulated and 47 downregulated) were identified as differentially expressed between tumor and normal samples and were also included in the 6267 DE genes (Fig. 1e, f).

### The network of TFs and genes

**Fig. 2:**
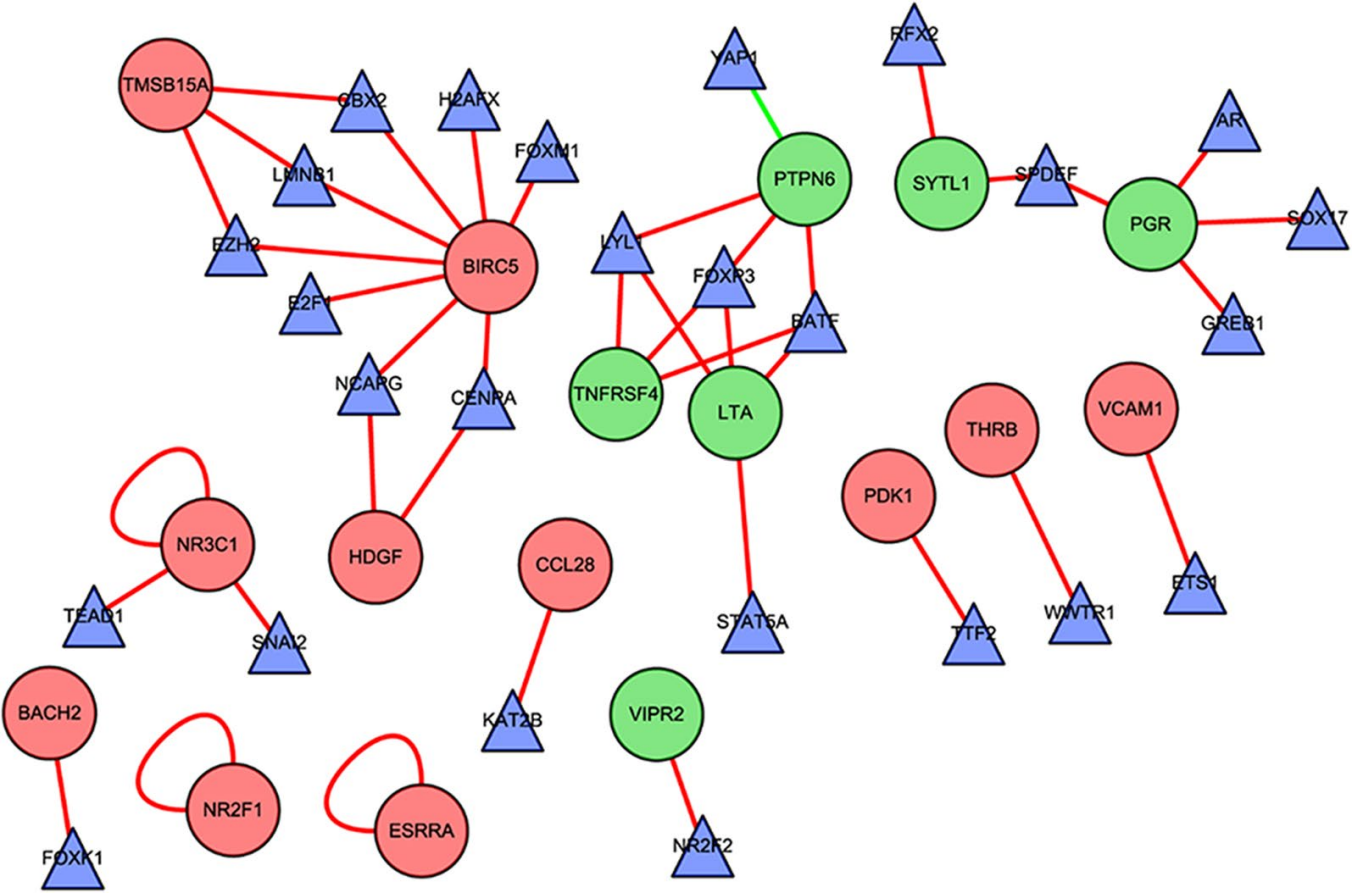
The network of differentially expressed transcription factors and immune-OS-related genes. The red balls indicated the upregulated genes and the green balls indicated the downregulated genes associated with immune and survival. The triangles are differentially expressed transcription factors. Red or green line and semicircle indicated positive or negative correlation individually

To enhance the understanding of the potential function of DE TFs and prognosis-related DE immune genes in EC, we constructed a TF and gene network based on the coexpression method. The interaction relationships are listed in Additional file 5: Table S2. The interactional network is shown in Fig. 2.

### GO and KEGG analysis of TFs and immune-related genes

**Fig. 3:**
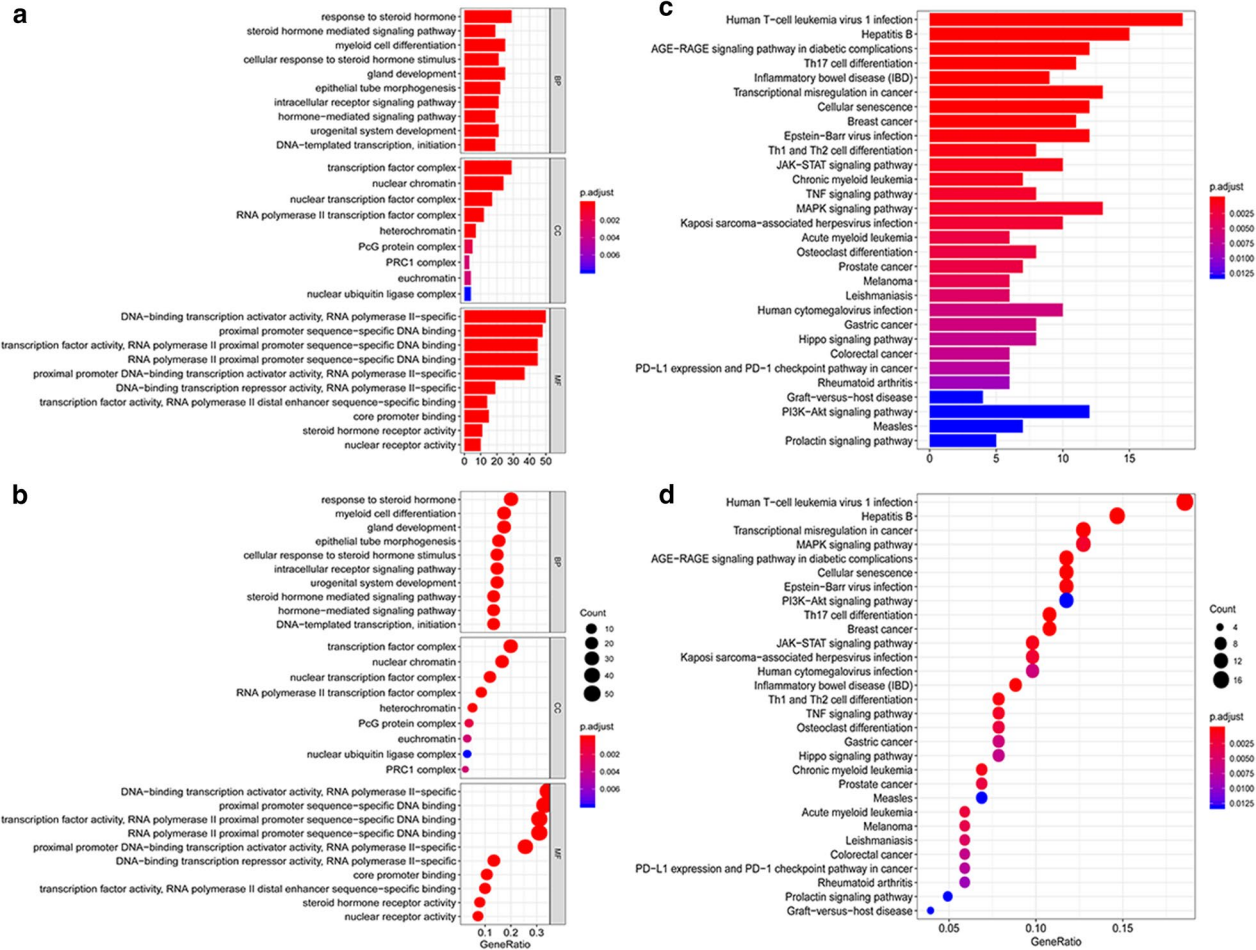
Functional enrichment analysis of DE TFs and immune-OS-related DE genes were accomplished by DAVID and KOBAS. **a**, **b** Gene Ontology enriched biological functions. **c**, **d** The enriched KEGG pathways

The study indicated that there were 29 GO terms and 30 enrichment pathways. Some tumor-related regulatory pathways were observed, including PD-L1 expression and PD-1 checkpoint pathway, MAPK pathway, PI3–Akt pathway and so on. The enrichment analysis results demonstrated the enrichment of regulatory functions, such as transcription factor complex, nuclear chromatin, and DNA-binding transcription activator activity. The GO term results in EC were shown in Fig. 3a, b, and the significantly enriched KEGG pathways in EC were shown in Fig. 3c, d.

### Identification of prognostic lncRNAs

**Fig. 4:**
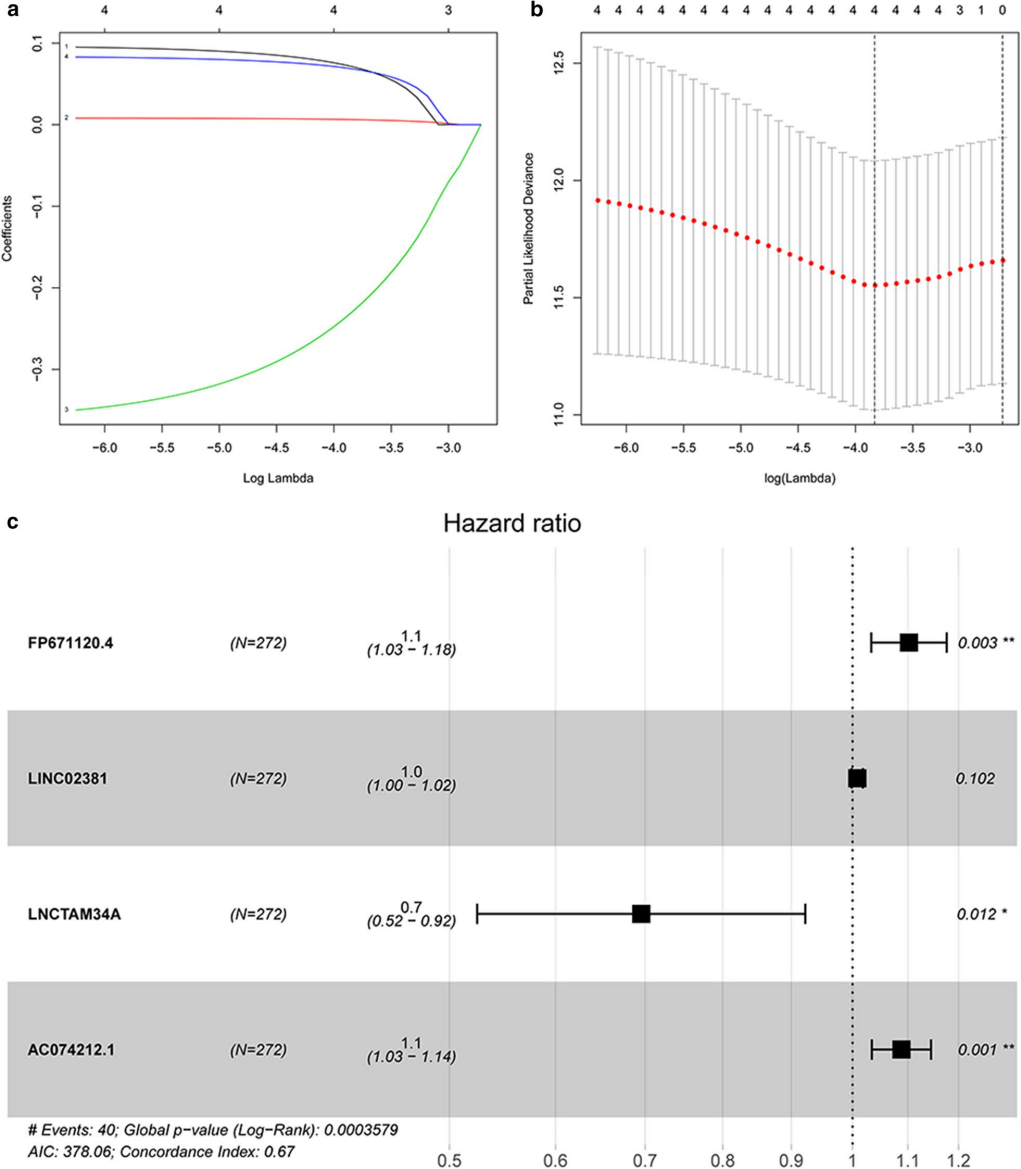
Identification of immune related prognostic lncRNA. **a**, **b** Least absolute shrinkage and selection operator analysis (LASSO) parameter adjustment and lambda profiles of 4 lncRNA selected by univariate Cox regression analysis. **c** Multivariate Cox regression analysis of 4 lncRNA. lncRNA, long noncoding RNA

A total of 204 lncRNAs that may be related to immunity were explored. Then, we divided 541 patients into the training set and testing set by the complete randomization method. By univariate Cox regression analysis in the training set, 4 immune-associated lncRNAs were identified to correlate with OS (P < 0.01). Then, a LASSO regression model was carried out to perform the next filtering of the 4 lncRNAs mentioned before. Glmnet from R software package was used for lasso regression analysis (iteration = 1000). The trajectory changes of the coefficients of four independent variables were presented in Fig. 4a. Furthermore, cross-validation was applied for model construction, as shown in Fig. 4b, indicating that the mean cross-validated error was minimal when λ = 0.0216. At this point, the 4 immune-related lncRNAs were confirmed to have close relativity with overall survival in EC. Afterwards, the result of multivariate Cox regression showed that among the four lncRNAs, three lncRNAs (FP671120.4, LINC02381 and AC074212.1) with positive coefficients may be poor prognostic indicators, while the remaining lncRNA(LNCTAM34A) could be a favorable prognostic factor (Fig. 4c).

### The 4-lncRNA signature for survival prediction

**Fig. 5:**
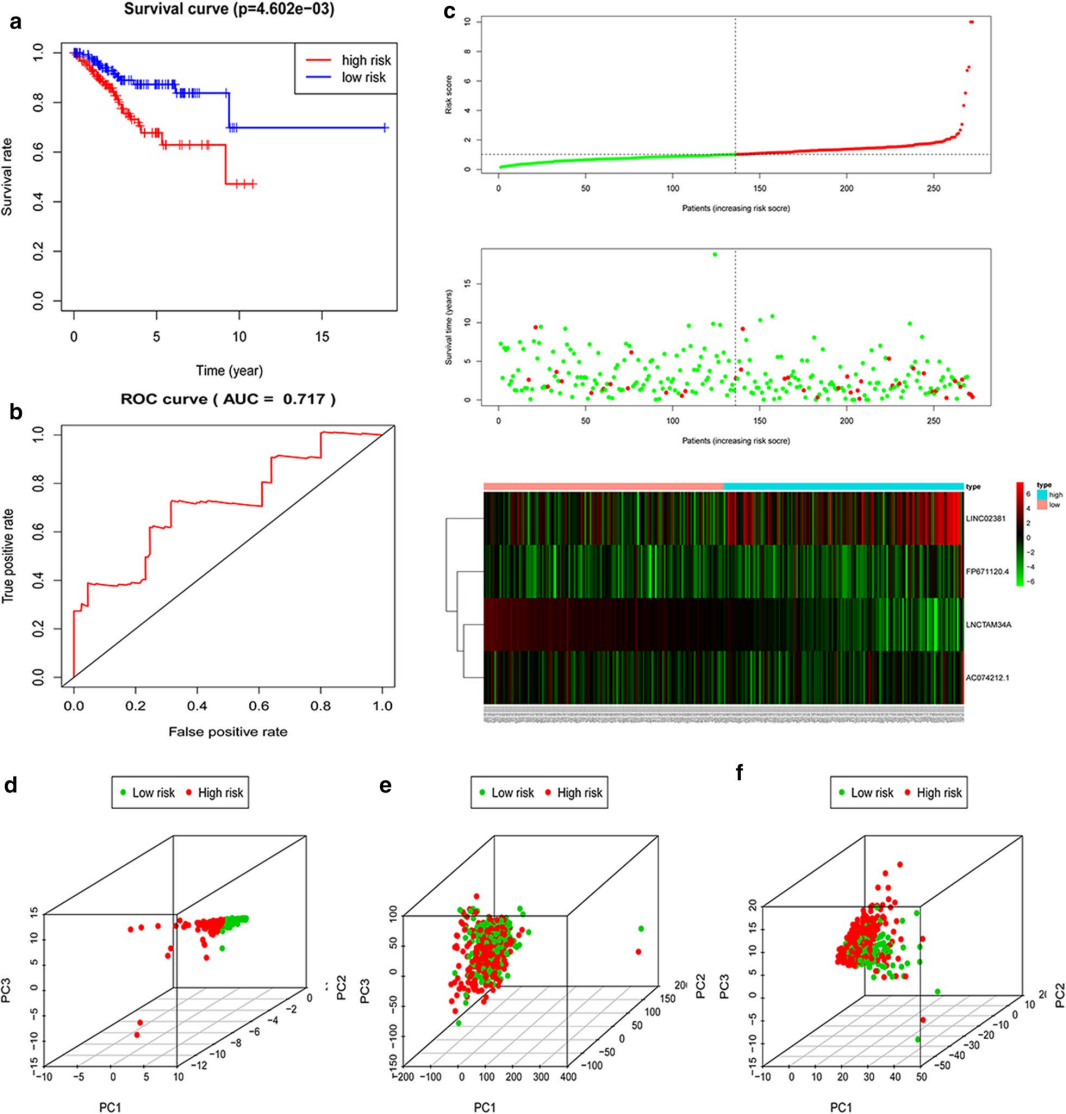
Construction of 4-lncRNA signature in the training set. **a** Kaplan–Meier survival curves revealed the difference in overall survival between high- and low-risk groups patients with EC. **b** Receiver operating characteristic (ROC). **c** The distribution of risk score, survival duration and expression profiles of 4-lncRNA in high- and low-risk groups. **d** Immune status analysis in high- and low-risk groups based on risk lncRNA by principal components analysis (PCA). **e**, **f** PCA based on the whole genome expression set and immune-related lncRNA set

In the training cohort, 272 samples were classified into a high-risk group (n = 136) and a low-risk group (n = 136) according to the calculated median cutoff value of the risk score. The Kaplan–Meier survival curve analysis revealed that patients with high-risk scores had an obviously poorer OS than those with low-risk scores (P = 4.602e−03, Fig. 5a). The AUC for the 4-lncRNA signature achieved 0.717 (Fig. 5b). The distribution of the risk score, survival duration of EC patients and the expression profiles plotted by risk heatmap of the 4 prognostic lncRNAs are demonstrated in Fig. 5c. The patients in the high-risk score group suffered poorer survival than patients in the low-risk score group.

The testing set and entire set were respectively divided into a high-risk group (n = 137 in the testing set, n = 273 in the entire set) and a low-risk group (n = 132 in the testing set, n = 268 in the entire set) according to the expression of the 4-lncRNA. The results showed that patients with high risk scores had poorer survival outcomes than patients with low risk scores (Additional file 6: Figure S4a, Additional file 7: Figure S5a). The AUC for the 4-lncRNA signature in the testing set and the entire set reached 0.686 and 0.703, respectively (Additional file 6: Figure S4b, Additional file 7: Figure S5b).

PCA was performed to detect the biological function of 4-lncRNA signature in EC, based on 4 lncRNAs in model, the whole genome expression set and immune-related lncRNA set (Fig. 5d–f). By using four lncRNAs in the signature and immune related lncRNAs, patients in low- and high-risk groups were separated into two different directions. It indicated that EC patients in low- and high-risk groups generally displayed in distinct immune status patterns, and the different immune states can be distinguished by the lncRNA signature.

### Assessment of independent risk factors

**Fig. 6:**
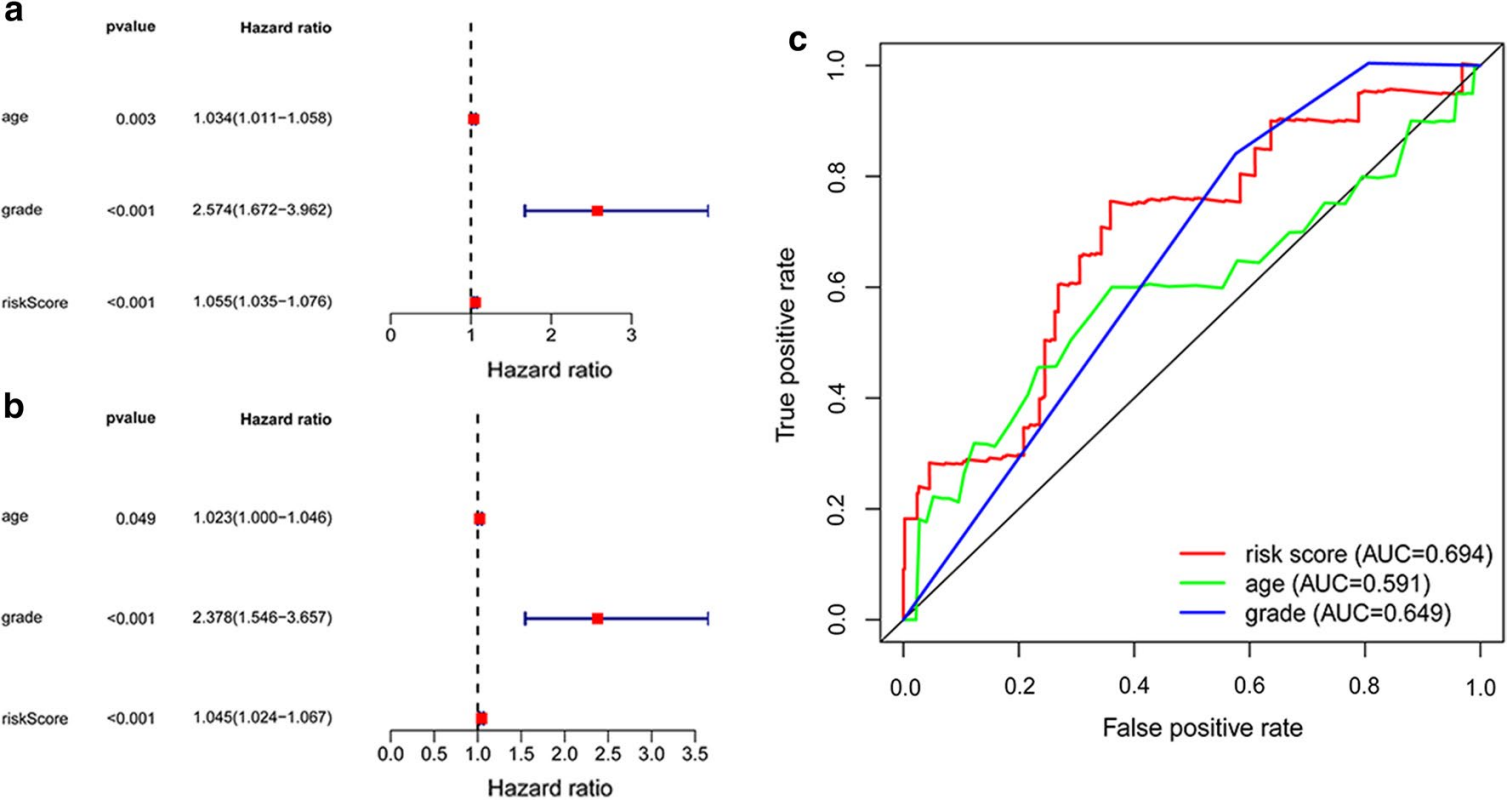
Assessment of independent risk factors. **a** Age, grade and risk score were the independent prognostic indicators by univariate analysis. **b** Age, grade and risk score were the independent prognostic indicators by multivariate analysis. **c** ROC curves showed the predict potential of 4-lncRNA signature

The estimation and verification of independent risk factors were conducted by Cox regression analyses. Univariate Cox regression analysis showed that age, histologic grade and risk score based on 4 immune-related lncRNA signature were identified as factors influencing survival (Fig. 6a). The multivariate Cox regression results demonstrated that the aforementioned features including age (HR = 1.023, P = 0.049), grade (HR = 2.378, P < 0.001) and risk score (HR = 1.045, P < 0.001), were all independent prognostic indicators of EC (Fig. 6b). Moreover, ROC curves were calculated to explore the prognostic forecast capabilities and accuracy of the above factors. As Fig. 6c shown, the 4-lncRNA signature associated with immunity displayed a better AUC (AUC = 0.694) and can serve as an effective index to independently predict prognosis.

To evaluate the predictive ability of the signature and obtain more satisfactory results, survival analysis was performed in the randomly regrouped training set (n = 272) and test set (n = 269). As Additional file 8: Figure S6 and Additional file 9: Figure S7 shown, age (P = 0.005), grade (P = 0.002) and risk score (P < 0.001) were directly related to the prognosis of patients in training set. And in testing set, only grade (P = 0.004) and risk score (P < 0.001) were related to prognosis. Multivariate Cox regression analysis indicated that aside from age (training set: P = 0.055, testing set: P = 0.177), only grade (training set: P = 0.004, testing set: P = 0.012) and risk score (training set: P = 0.002, testing set: P < 0.001) were statistically independent predictive indicators of endometrial cancer. The AUC for the risk score (training set: AUC = 0.733, testing set: AUC = 0.657) based on 4-lncRNA signature in both training set and testing set was higher than that for grade (training set: AUC = 0.665, testing set: AUC = 0.635) and age (training set: AUC = 0.651, testing set: AUC = 0.544). It indicated that 4-lncRNA had the ability to compete sufficiently with traditional clinical factors to predict OS of EC patients. The results demonstrated the superiority of 4-lncRNA in predicting HCC patient OS compared with classical clinical and pathological staging systems.

### Comparison of the immune-related lncRNA signature with other prognostic models

**Fig. 7:**
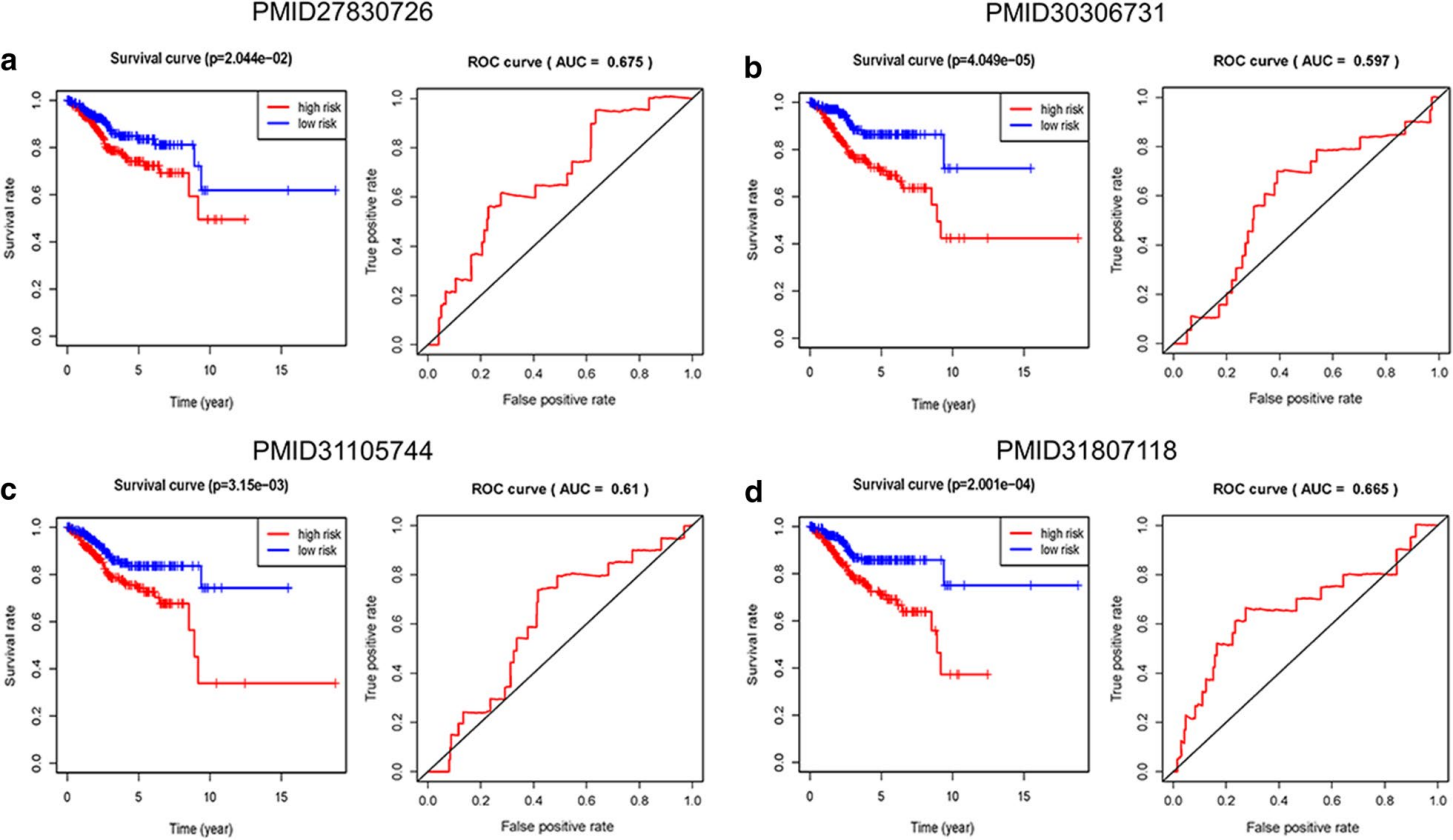
Four signatures predict prognosis in patients with endometrial cancer in the entire set. **a** PMID27830726. **b** PMID30306731. **c** PMID31105744. **d** PMID31807118

In order to determine whether this immune-related lncRNA signature had more superiority than other endometrial cancer prognostic biomarkers, we compared our signature with nine-gene signature [25], six-gene signature [26], seven-gene signature [27], and nine-gene signature [28]. The genes in these signatures were obtained from the literature, and we constructed the ROC curves and survival curves of the entire cohort. As shown on Fig. 7, the AUC values of OS in these models were 0.703, 0.675, 0.597, 0.61 and 0.665, respectively. Through analysis and comparison of these signatures, we know that the accuracy of our signature in predicting prognosis of endometrial cancer is higher than that of other four biomarkers (Table 2).

**Table 2:** Comparison of the immune-related lncRNA signature with four other prognostic models

Model	AUC	P-value
Immune-related lncRNA signature	0.703	6.902e−06
Nine-gene signature (PMID27830726)	0.675	2.044e−02
Six-gene signature (PMID30306731)	0.597	4.049e−05
Seven-gene signature (PMID31105744)	0.61	3.15e−03
Nine-gene signature (PMID31807118)	0.665	2.001e−04

### Prognostic value of each of the four lncRNAs

**Fig. 8:**
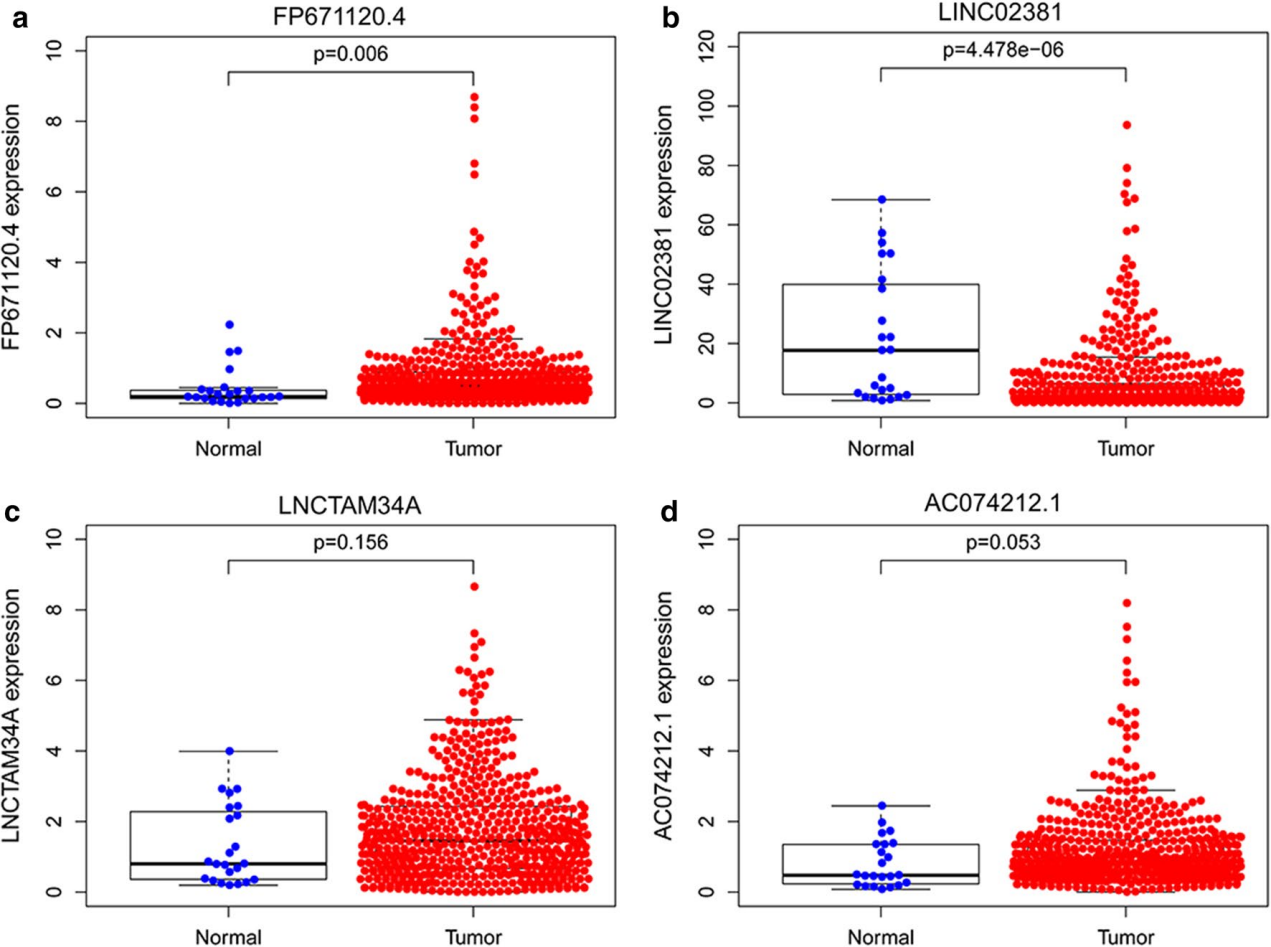
The comparison of immune-related lncRNA expression between EC tissues and normal tissues. **a** FP671120.4. **b** LINC02381. **c** LNCTAM34A. **d** AC074212.1

**Fig. 9:**
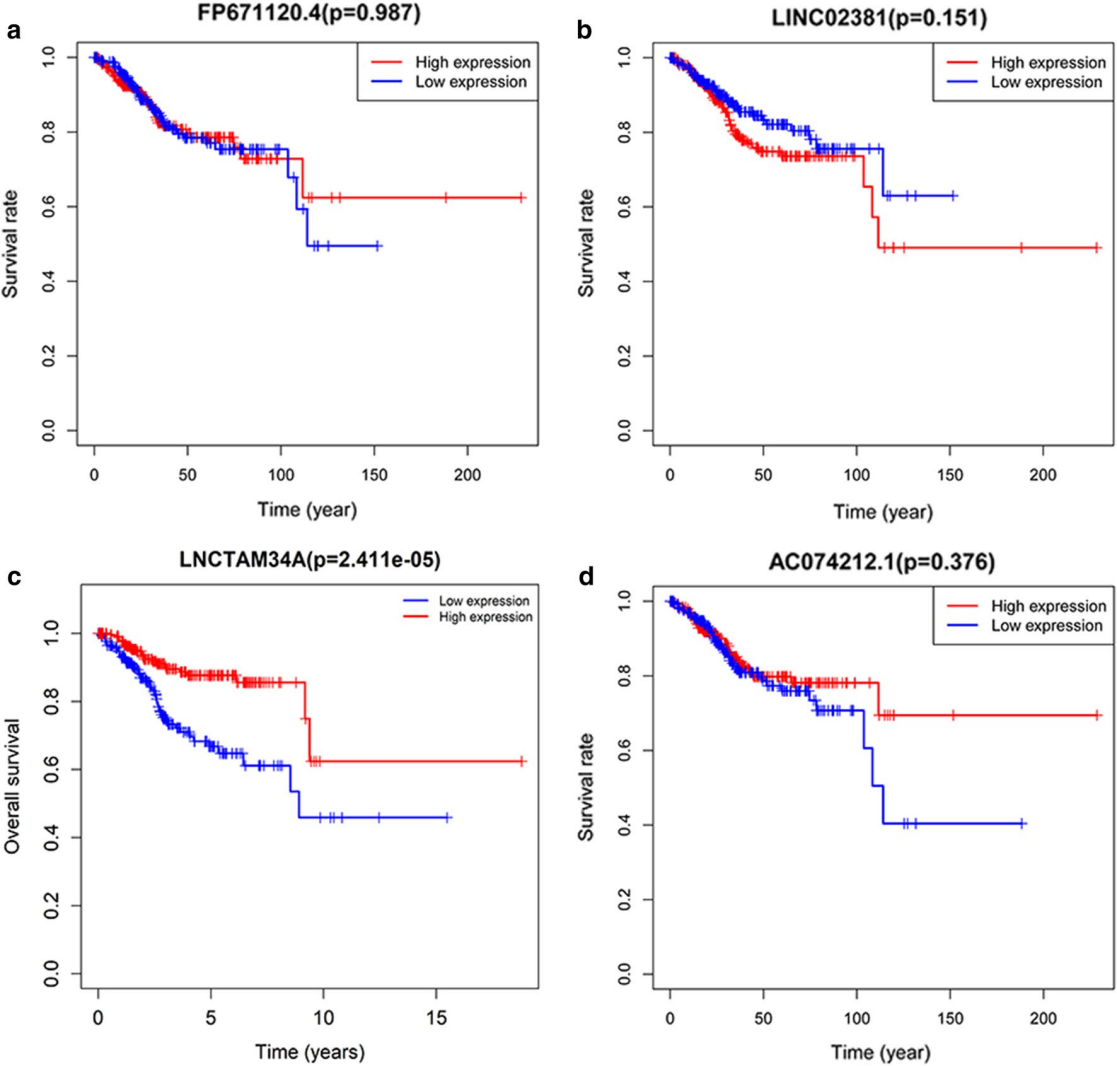
The Kaplan–Meier survival of four immune-related lncRNAs respectively. **a** FP671120.4. **b** LINC02381. **c** LNCTAM34A. **d** AC074212.1

We compared the corresponding expression levels of each of the four lncRNAs (FP671120.4, LINC02381, LNCTAM34A and AC074212.1) between EC tissues and non-tumor tissues (Fig. 8). Finally, a total of 541 EC patients were divided into the high- and low-expression groups by utilizing the median expression level of each lncRNA as the critical value. Kaplan–Meier survival analysis was employed to explore the prognostic capacity of each lncRNA, and the analysis results are presented in Fig. 9.

### QRT-PCR verification

**Fig. 10:**
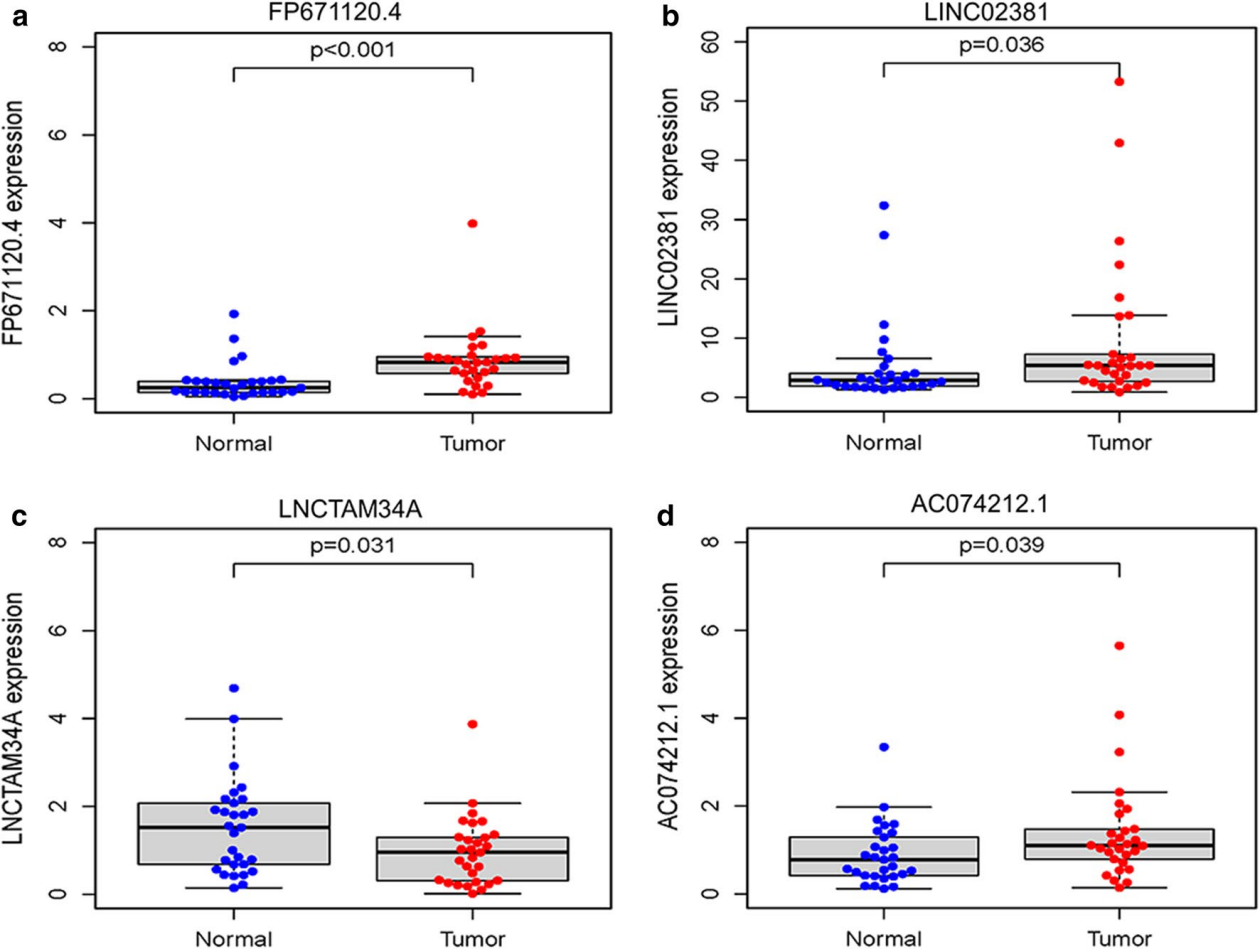
QRT‐PCR validation of immune-related lncRNA expression between EC tissues and normal tissues of 29 patients. **a** FP671120.4. **b** LINC02381. **c** LNCTAM34A. **d** AC074212.1

**Fig. 11:**
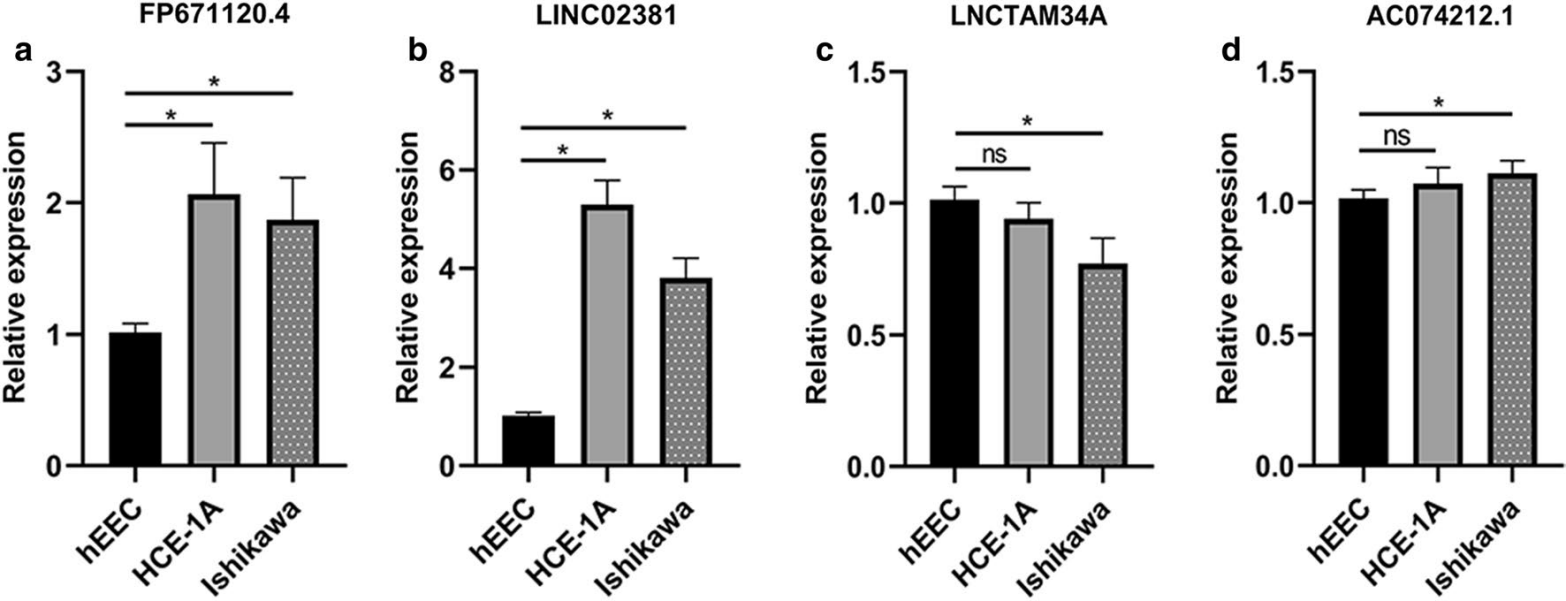
Relative expression of lncRNA in EC cells in endometrial epithelial cell line hEEC and EC cell lines HEC-1A and Ishikawa. **a** FP671120.4. **b** LINC02381. **c** LNCTAM34A. **d** AC074212.1. *P < 0.05 compared to the normal group. ns: no statistical significance compared to the normal group

**Fig. 12:**
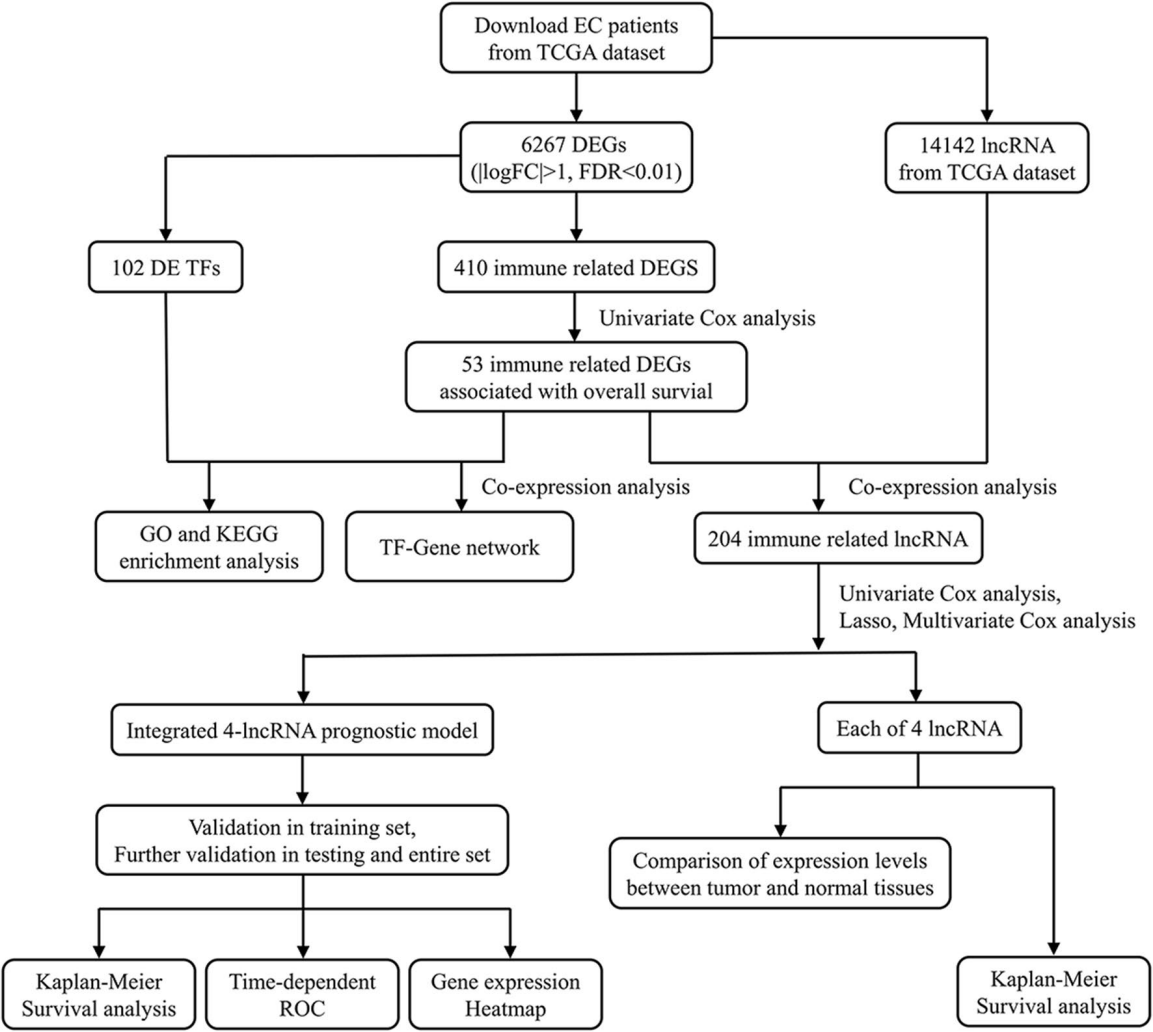
The flowchart of our research strategy

In order to further evaluate the reliability of the immune-related signature, we measured the actual expression of four lncRNAs in the tissues of 29 patients by qRT-PCR. Compared with adjacent normal tissues, FP671120.4, LINC02381 and AC074212.1 were upregulated while LNCTAM34A were downregulated in EC tissues (Fig. 10). Similarly, compared with normal endometrial epithelial cell line hEEC, the expression level of FP671120.4 and LINC02381 significantly upregulated in EC cell lines (Fig. 11). In addition, AC074212.1 expression was upregulated and LNCTAM34A was downregulated in Ishikawa. However, the expression of AC074212.1 and LNCTAM34A were both no significant difference between HEC-1A and hEEC. The results of qRT-PCR verification in 29 patients with endometrial cancer and in cells were consistent with the above-mentioned bioinformatics results. It revealed the validity and reliability of the biological signature we constructed. The flowchart of our research strategy is described in Fig. 12.

## Discussion

Endometrial cancer ranks as the fifth most common cause of cancer death in the United States, increasingly, threatening female patients’ lives [29]. The discovery of objective and susceptible indicators is crucial to optimize clinical diagnosis and has instructive significance in judging the prognosis of EC accurately.

LncRNAs have been confirmed to play a key role in numerous pathological courses and remain stable in blood circulation, hence, lncRNAs are deemed biomarkers, offering guidance for therapy and determining eventual results in various carcinomas [30]. Although the abnormal expression of several lncRNAs in endometrial and ovarian cancer has been revealed in prior studies, realizing a comprehensive probe on this topic in gynecological cancers still needs long way off [31]. Pan et al. pointed out a two-way feedback ring composed of LINC01016 and miRNA that mediated cell biology phenotype transformation in EC [32]. Simultaneously, various lncRNAs have been indicated to participate in adjusting the immune system [33]. The immune response exerts a great impact on the pathophysiology and progression of solid tumors, including endometrial carcinoma. Likewise, immunotherapy, an innovative therapy modality with great promise, has recently been reported as a research hotspot [34]. Moreover, the immunoregulatory mechanism determines the occurrence and intensity of the immune response, and many transcription factors act as the main regulators to regulate the immune response process. Zaiss discussed the core role undertaken by Forkhead box transcription factors (FTFs) in the regulation of immune responses and homeostasis [19]. It is also worth noting that transcription factors and lncRNA can interact to promote appropriate regulation of gene expression [35, 36]. For instance, transcription factor SOX2 activates LINC01561 and promotes the proliferation by modulating SHCBP1 in NSCLC [37]. LncRNA FLICR negatively regulates transcription factor Foxp3, and the modulation may be associated with infections or tumors both related with increased Treg activity [38]. Meanwhile, the role of transcription factors in the regulation of inflammation has been increasingly reported. NF-kappa B controls the expression of multiple genes in endothelial cells at the site of inflammatory response, thus involving in infection and inflammation [39]. And Delpoux proposed Foxo1 has a key function in T cell differentiation and transport, thereby controlling the response of central memory CD8 T cells to infection [40]. In addition, years of research have adequately established the immense modulatory potential of transcription factor activity as a trigger of cancer, such as the transcription factors SNAIL1 and ZEB1, mediating the epithelial-to-mesenchymal transition-related signaling pathway [41]. Kingwell et al. concluded that elF4F promotes the expression capacity of the transcription factor of STAT1, thus potentiating the immune escape of melanoma [42].

In this study, we constructed a network in EC by making use of the TCGA database to explore the interactions between DE TFs and immune-OS-related genes. There were 6267 differentially expressed genes between tumor tissues and normal tissues obtained from the TCGA. Afterwards, the network of 29 DE TFs and 17 immune-OS-related genes was established on the basis of the results from the coexpression analysis. Furthermore, by conducting enrichment analyses, we explored 102 DE TFs and 53 DE genes associated with OS and the immune system, which were both included in the 6267 DE genes. The results of GO functional analysis and KEGG enrichment analysis revealed that the DE genes are significantly clustered in the transcription factor complex, DNA-binding transcription activator activity, intracellular receptor signaling pathway, MAPK signaling pathway, PI3K-AKT signaling pathway, and PD-L1 and PD-1 checkpoint pathway, which also accounted for a major portion of the enrichment characteristics. Among them, the PTEN/PI3K/AKT/mTOR pathway is the main signaling pathway participating in the metastasis of EC [43].

Moreover, we detected the association between 204 immune-related lncRNAs and EC patient survival prognosis by performing a series of analysis processes, such as univariate, LASSO and multivariate Cox analyses. The 4 lncRNAs, FP671120.4, LINC02381, LNCTAM34A and AC074212.1, showed notable prognostic value for EC patients in the training dataset. Next, the 4 immune-relevant lncRNAs were integrated by adopting risk scoring methods, and the results suggested that the signature could forecast patient survival independently. Furthermore, we verified the accuracy of the signature’s prognostic value in the testing dataset and the entire dataset, and the results demonstrated that the development of the 4-lncRNA signature model was successful in both high-level robustness and improved repeatability in two key aspects. Our research showed that the immune score is closely linked with adverse outcomes in patients diagnosed with endometrial cancer. In addition, in accordance with univariate and multivariate analyses results, the 4-lncRNA signature was further proven to serve as an independent predictor of endometrial cancer survival prognosis. Compared with the age and grade curve, the four lncRNA signature ROC curve displayed a greater AUC. Therefore, we infer that it is reasonable to consider immune-related lncRNA that is independent of other traditional clinical features as beneficial in terms of the accuracy of measuring the prognosis of EC patients. Previous studies have constructed other prognostic models of endometrial cancer [25–28]. By comparing these models, we confirmed the prognostic ability of our immune-related lncRNA signature was higher than that of previous gene signature. It indicated that our four-lncRNA signature was better indicator for making prognosis assessment of endometrial cancer. In addition, we quantitatively analyzed these four lncRNAs by conventional qRT-PCR, and the results were consistent with the above-mentioned bioinformatics results. Therefore, the biological signature we constructed can be identified as a valuable and reliable predictor.

In subsequent steps, to provide better application to clinical diagnosis, we estimated the expression differences of individual lncRNAs among the 4-lncRNA signature between carcinoma tissues and normal tissues and performed KM survival analysis for each of the four lncRNAs. The results showed that patients with high LNCTAM34A expression had a longer survival time than those with low expression, while there was no significant difference in LNCTAM34A expression between cancer and normal tissues, perhaps because the data sample we used was limited. In addition, LNCTAM34A has been found to mediate the high expression of miR34a to protect cells from outside stress stimuli [44]. Moreover, the expression results of two genes in PCR were not identical to each of those analyzed in database. It may be due to the small sample size of normal endometrial tissue in the database or heterogeneity of the tumor.

Nevertheless, there remain certain limitations to our study. To solve the problem of insufficient samples, our signature should be adequately validated in other databases and in studies with larger amounts of endometrial cancer data. The major research analysis approach we employed is bioinformatics technology, which has emerged as an effective and reliable tool, while the functional mechanism and interaction network of lncRNAs are complicated. Further functional studies on the lncRNAs we explored, the acquisition of additional experimental data (in vitro and in vivo) and long-term follow-up observations are essential to estimate the accuracy of our signature and confirm our findings.

## Conclusions

In summary, our team proposed a 4-lncRNA signature based on the immune system as a possible underlying biomarker for effective diagnosis and prognosis assessment. This signature could be considered a novel target of an immunological approach for endometrial cancer with a bright perspective (Additional file 8: Figure S7, Additional file 9: Table S2).

## Supplementary information

**Additional file 1: Figure S1.** The calculation formula of risk score.

**Additional file 2: Figure S2.** Statement on informed consent.

**Additional file 3: Table S1.** Primers applied in qPCR**.**

**Additional file 4: Figure S3.** Identification of immune related and differentially expressed genes associated with overall survival (OS) of EC patients. The forest plot showed 53 out of 410 immune-related DE genes are related to survival.

**Additional file 5: Table S2.** Co-expression analysis of DE TFs and immune-OS-related DE genes. The co-expression analysis result displays that there were interactions between 29 DE TFs and 17 immune-OS-related DE genes.

**Additional file 6: Figure S4.** Verification of the signature in the testing set. (a) Kaplan–Meier survival analysis between high- and low-risk groups patients with EC. (b) Receiver operating characteristic (ROC). (c) The distribution of risk score, survival duration and expression profiles of 4-lncRNA in high- and low-risk groups.

**Additional file 7: Figure S5.** Verification of the signature in the entire set. (a) Kaplan–Meier survival analysis between high- and low-risk groups patients with EC. (b) Receiver operating characteristic (ROC). (c) The distribution of risk score, survival duration and expression profiles of 4-lncRNA in high- and low-risk groups.

**Additional file 8: Figure S6.** Assessment of independent risk factors in training set. (a) Age, grade and risk score were the independent prognostic indicators by univariate analysis. (b) Grade and risk score were the independent prognostic indicators by multivariate analysis. (c) ROC curves showed the predict potential of 4-lncRNA signature.

**Additional file 9: Figure S7.** Assessment of independent risk factors in testing set. (a) Grade and risk score were the independent prognostic indicators by univariate analysis. (b) Grade and risk score were the independent prognostic indicators by multivariate analysis. (c) ROC curves showed the predict potential of 4-lncRNA signature.

〹

## Data Availability

The data supporting the findings of this study are available from the corresponding author upon request.

## Abbreviations

EC: Endometrial cancer
ROC: Receiver operating characteristic
TCGA: The Cancer Genome Atlas
DE: Differential expression
TF: Transcription factor
DEG: Differentially expressed gene
OS: Overall survival
PCA: Principal component analysis
