# Supplementary figures and images for: Integrated analysis of immune-related genes in endometrial carcinoma
Source: Cancer Cell Int. 2020 Oct 2;20:477. doi: 10.1186/s12935-020-01572-6 (PMC7531161; doi:10.1186/s12935-020-01572-6)

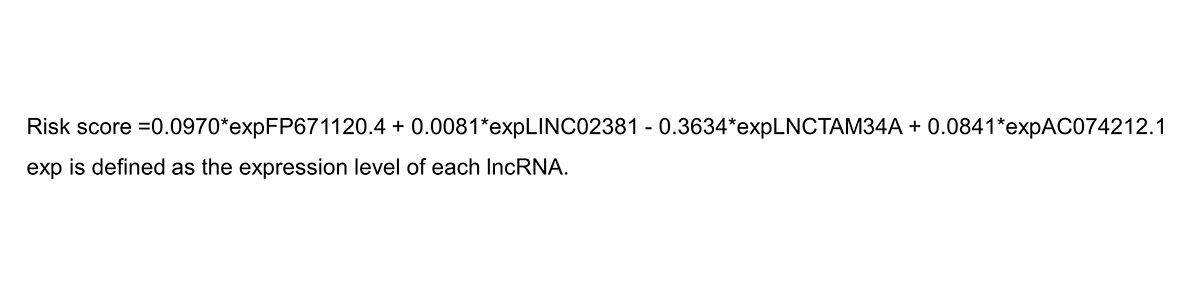

Supplement: Supplementary file 1 — Additional file 1: Figure S1. The calculation formula of risk score. [file 12935_2020_1572_MOESM1_ESM.tif]

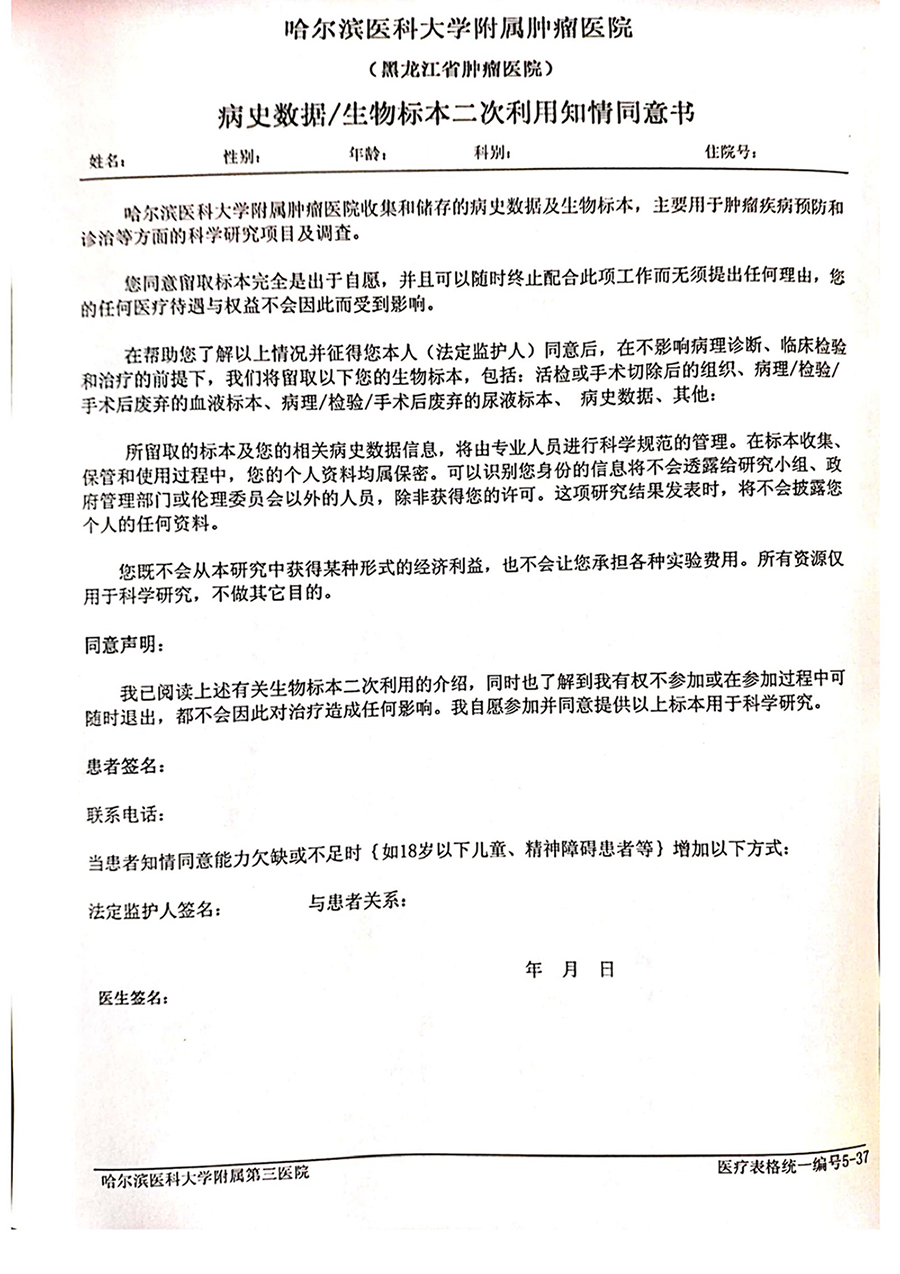

Supplement: Supplementary file 2 — Additional file 2: Figure S2. Statement on informed consent. [file 12935_2020_1572_MOESM2_ESM.tif]

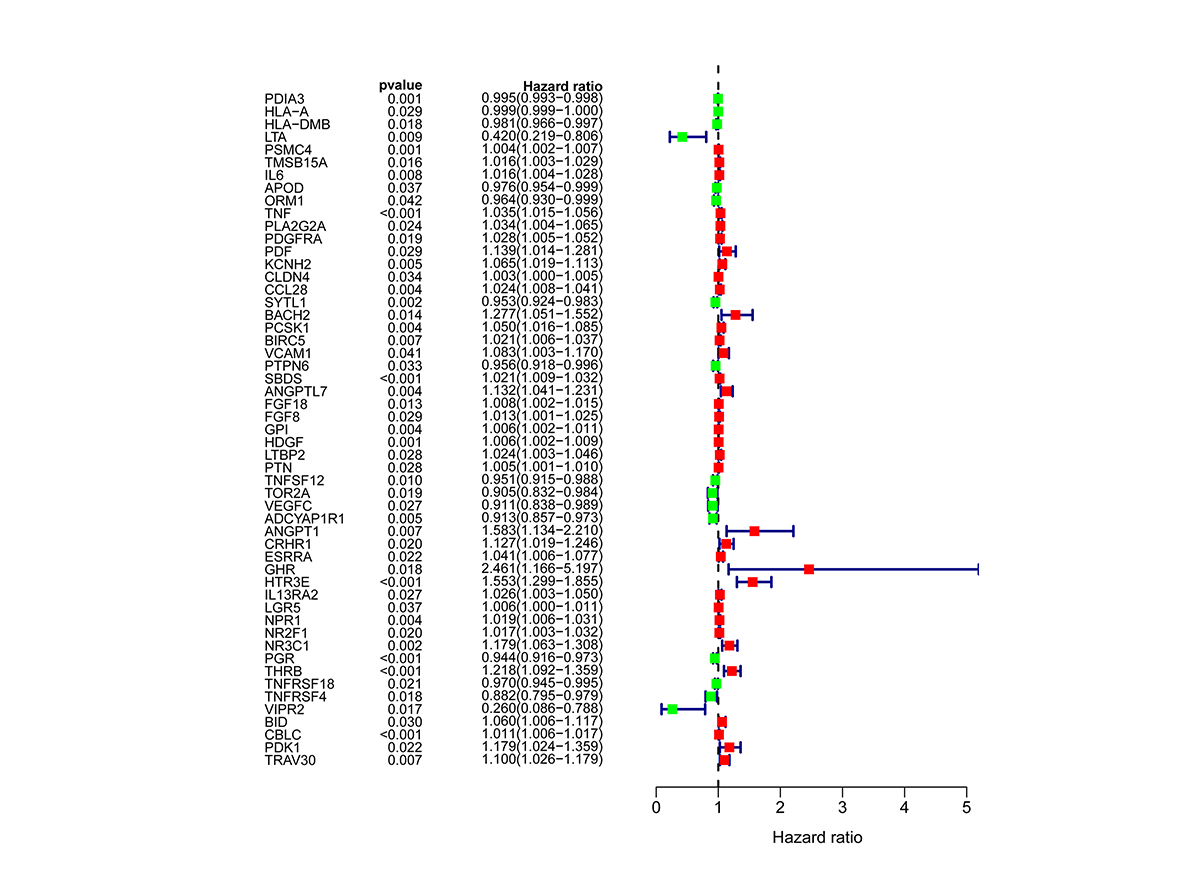

Supplement: Supplementary file 4 — Additional file 4: Figure S3. Identification of immune related and differentially expressed genes associated with overall survival (OS) of EC patients. The forest plot showed 53 out of 410 immune-related DE genes are related to survival. [file 12935_2020_1572_MOESM4_ESM.tif]

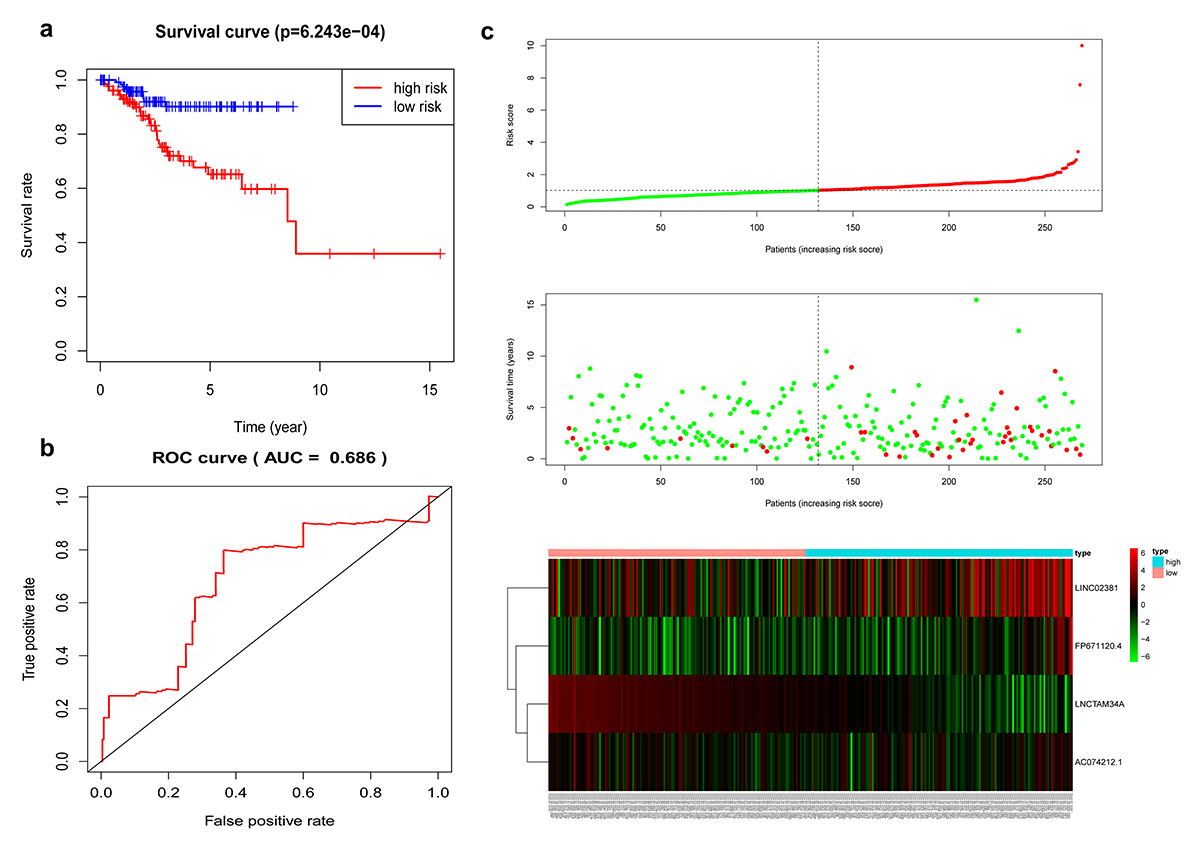

Supplement: Supplementary file 6 — Additional file 6: Figure S4. Verification of the signature in the testing set. (a) Kaplan–Meier survival analysis between high- and low-risk groups patients with EC. (b) Receiver operating characteristic (ROC). (c) The distribution of risk score, survival duration and expression profiles of 4-lncRNA in high- and low-risk groups. [file 12935_2020_1572_MOESM6_ESM.tif]

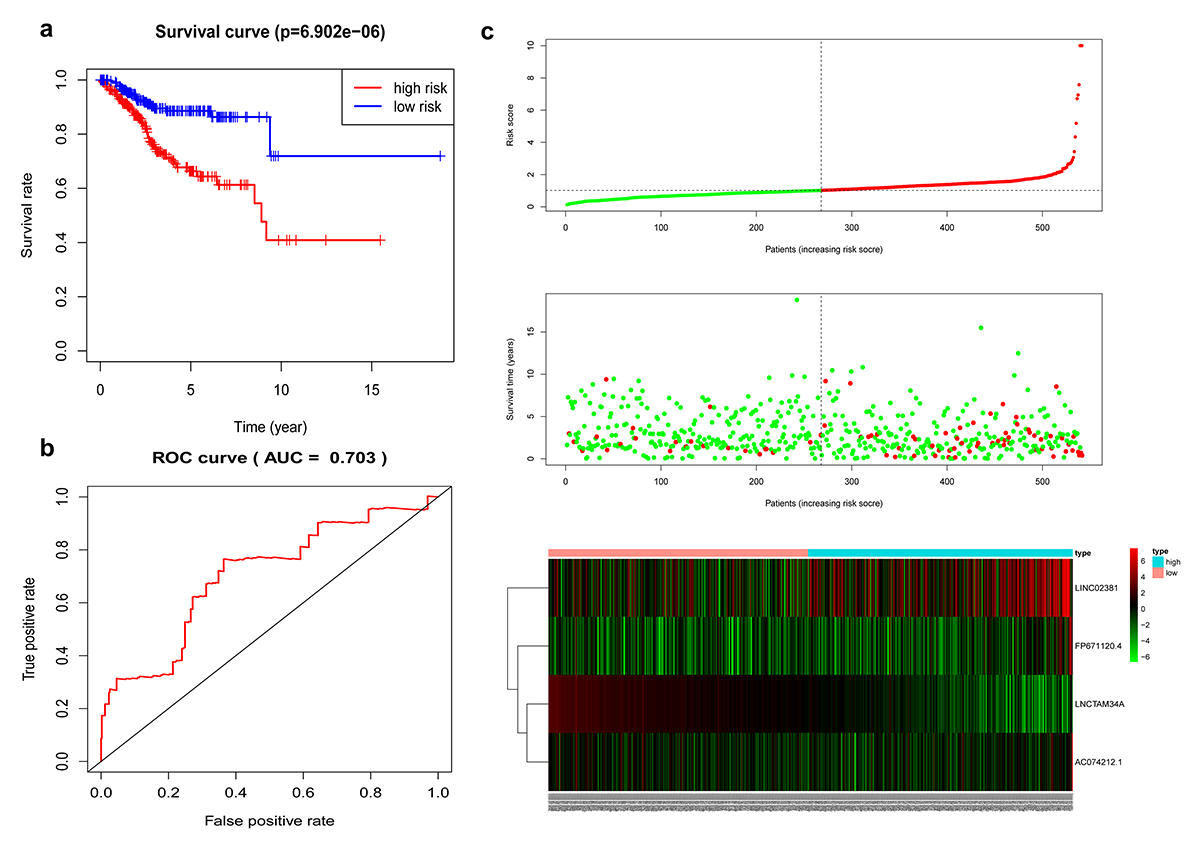

Supplement: Supplementary file 7 — Additional file 7: Figure S5. Verification of the signature in the entire set. (a) Kaplan–Meier survival analysis between high- and low-risk groups patients with EC. (b) Receiver operating characteristic (ROC). (c) The distribution of risk score, survival duration and expression profiles of 4-lncRNA in high- and low-risk groups. [file 12935_2020_1572_MOESM7_ESM.tif]

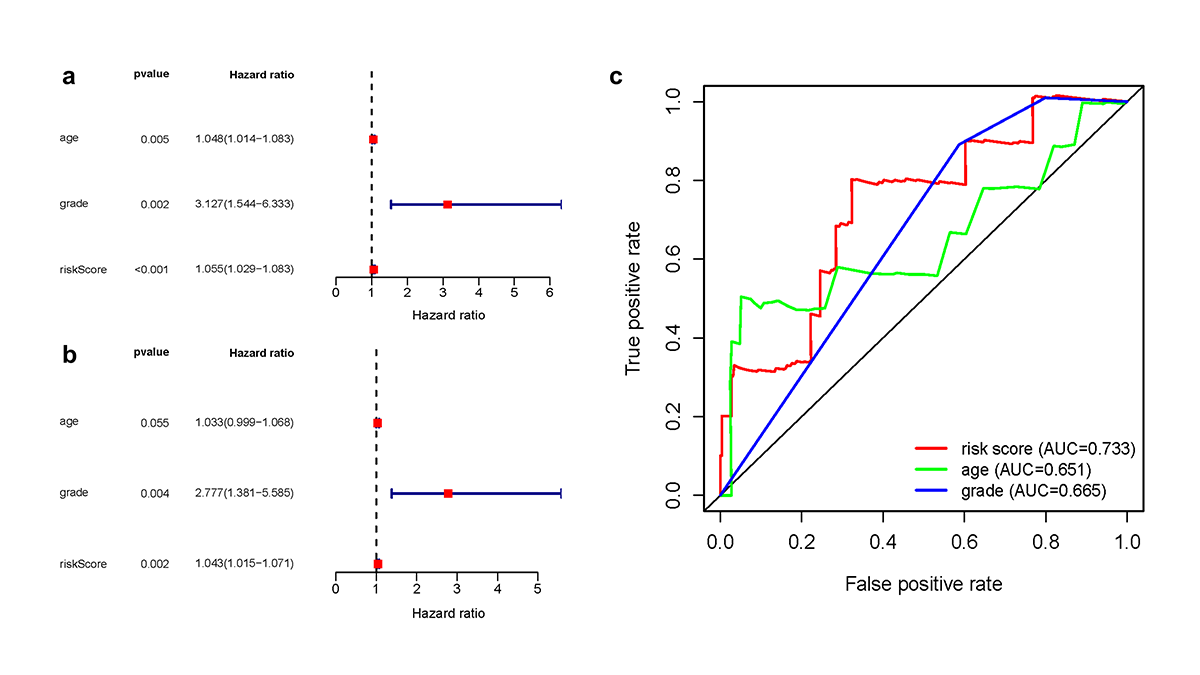

Supplement: Supplementary file 8 — Additional file 8: Figure S6. Assessment of independent risk factors in training set. (a) Age, grade and risk score were the independent prognostic indicators by univariate analysis. (b) Grade and risk score were the independent prognostic indicators by multivariate analysis. (c) ROC curves showed the predict potential of 4-lncRNA signature. [file 12935_2020_1572_MOESM8_ESM.tif]

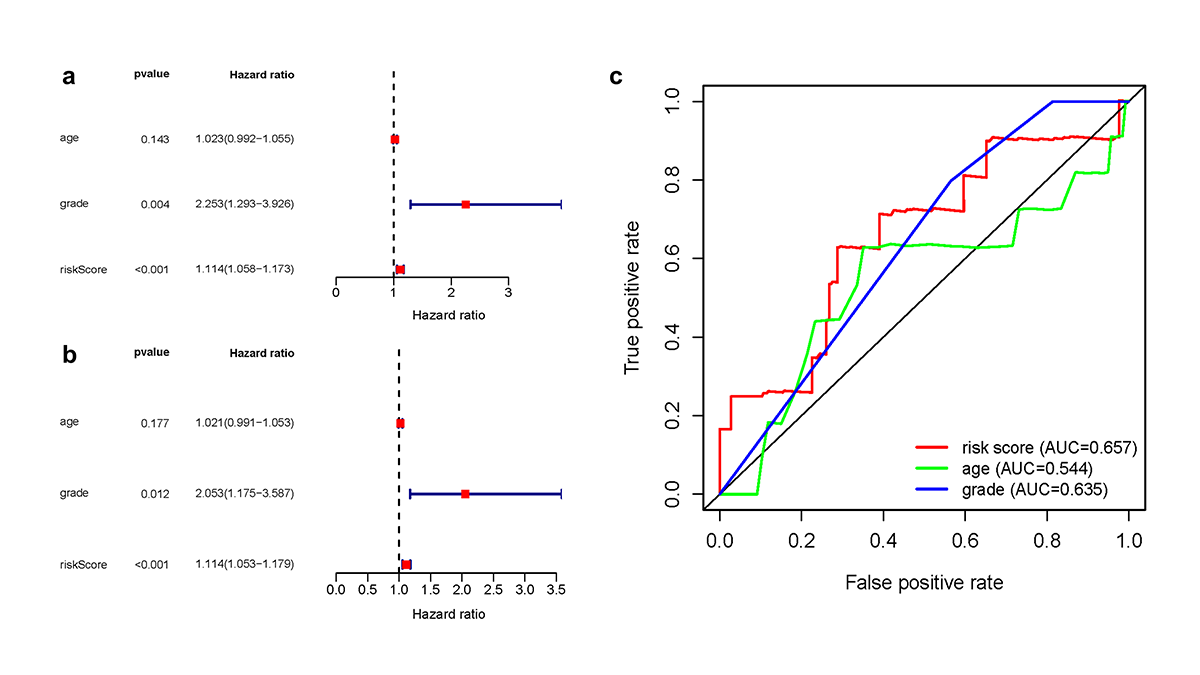

Supplement: Supplementary file 9 — Additional file 9: Figure S7. Assessment of independent risk factors in testing set. (a) Grade and risk score were the independent prognostic indicators by univariate analysis. (b) Grade and risk score were the independent prognostic indicators by multivariate analysis. (c) ROC curves showed the predict potential of 4-lncRNA signature. [file 12935_2020_1572_MOESM9_ESM.tif]
