# Supplementary material for: Integrated analysis of immune-related genes in endometrial carcinoma
Source: Cancer Cell Int. 2020 Oct 2;20:477. doi: 10.1186/s12935-020-01572-6 (PMC7531161; doi:10.1186/s12935-020-01572-6)
Supplement: Supplementary file 3 — Additional file 3: Table S1. Primers applied in qPCR. [file 12935_2020_1572_MOESM3_ESM.docx]

Table S1. Primers applied in qPCR

| Gene | Forward Primer (5’-3’) | Reverse Primer (5’-3’) |
| --- | --- | --- |
| FP671120.4 | CGCGTCTGGCCATAACATCT | ACCCTGGGCGACAAGAGTG |
| LINC02381 | TGGCAGGATGGAAATCTAGATGA | TGCTCACCAAGGTCTGGAAC |
| LNCTAM34A | CGGCACTGGGAGAAGACGA | TGAATGCTCAGGCATCTGATCTG |
| AC074212.1 | CACCTTCGGATTCCAGGAGTT | CCTCCACTTGGTACTAGCTGTAAGC |
| β-actin | TGACCAGCCGACACCAAGA | GCACGAACAAGCAACTGAACT |
