# Supplementary material for: Integrated analysis of immune-related genes in endometrial carcinoma
Source: Cancer Cell Int. 2020 Oct 2;20:477. doi: 10.1186/s12935-020-01572-6 (PMC7531161; doi:10.1186/s12935-020-01572-6)
Supplement: Supplementary file 5 — Additional file 5: Table S2. Co-expression analysis of DE TFs and immune-OS-related DE genes. The co-expression analysis result displays that there were interactions between 29 DE TFs and 17 immune-OS-related DE genes. [file 12935_2020_1572_MOESM5_ESM.docx]

Table S2. Co-expression analysis of DE TF and immune-OS-related DE genes

| TF | Immune Gene | Coef | pvalue | Regulation |
| --- | --- | --- | --- | --- |
| AR | PGR | 0.464749 | 2.43E-30 | postive |
| BATF | LTA | 0.68936 | 1.70E-77 | postive |
| BATF | PTPN6 | 0.502413 | 6.07E-36 | postive |
| BATF | TNFRSF4 | 0.614284 | 1.98E-57 | postive |
| CBX2 | TMSB15A | 0.422658 | 7.47E-25 | postive |
| CBX2 | BIRC5 | 0.561775 | 2.52E-46 | postive |
| CENPA | BIRC5 | 0.564502 | 7.48E-47 | postive |
| CENPA | HDGF | 0.545727 | 2.60E-43 | postive |
| E2F1 | BIRC5 | 0.506725 | 1.25E-36 | postive |
| ESRRA | ESRRA | 0.997378 | 0 | postive |
| ETS1 | VCAM1 | 0.480183 | 1.49E-32 | postive |
| EZH2 | TMSB15A | 0.531872 | 7.74E-41 | postive |
| EZH2 | BIRC5 | 0.409496 | 2.74E-23 | postive |
| FOXK1 | BACH2 | 0.4261 | 2.84E-25 | postive |
| FOXM1 | BIRC5 | 0.44087 | 3.91E-27 | postive |
| FOXP3 | LTA | 0.785616 | 1.79E-114 | postive |
| FOXP3 | PTPN6 | 0.501907 | 7.30E-36 | postive |
| FOXP3 | TNFRSF4 | 0.587152 | 1.94E-51 | postive |
| GREB1 | PGR | 0.548959 | 6.63E-44 | postive |
| H2AFX | BIRC5 | 0.423146 | 6.51E-25 | postive |
| KAT2B | CCL28 | 0.433477 | 3.43E-26 | postive |
| LMNB1 | TMSB15A | 0.427295 | 2.02E-25 | postive |
| LMNB1 | BIRC5 | 0.435003 | 2.20E-26 | postive |
| LYL1 | LTA | 0.586109 | 3.21E-51 | postive |
| LYL1 | PTPN6 | 0.432103 | 5.10E-26 | postive |
| LYL1 | TNFRSF4 | 0.637929 | 3.78E-63 | postive |
| NCAPG | BIRC5 | 0.495467 | 7.41E-35 | postive |
| NCAPG | HDGF | 0.423176 | 6.46E-25 | postive |
| NR2F1 | NR2F1 | 0.999884 | 0 | postive |
| NR2F2 | VIPR2 | 0.469231 | 5.68E-31 | postive |
| NR3C1 | NR3C1 | 0.99572 | 0 | postive |
| RFX2 | SYTL1 | 0.413385 | 9.60E-24 | postive |
| SNAI2 | NR3C1 | 0.425067 | 3.80E-25 | postive |
| SOX17 | PGR | 0.464763 | 2.42E-30 | postive |
| SPDEF | SYTL1 | 0.441994 | 2.80E-27 | postive |
| SPDEF | PGR | 0.472732 | 1.80E-31 | postive |
| STAT5A | LTA | 0.431175 | 6.66E-26 | postive |
| TEAD1 | NR3C1 | 0.402912 | 1.56E-22 | postive |
| TTF2 | PDK1 | 0.433202 | 3.71E-26 | postive |
| WWTR1 | THRB | 0.454724 | 5.79E-29 | postive |
| YAP1 | PTPN6 | -0.41125 | 1.71E-23 | negative |
